# Official Account of the Exhumation of the Body of Hahnemann at the Cemetery Montmartre, and Its Removal to Père Lachaise

**Published:** 1898-08

**Authors:** 


THE
### HOMŒOPATHIC PHYSICIAN,
A MONTHLY JOURNAL OF
HOMŒOPATHIC MATERIA MEDICA AND CLINICAL MEDICINE.

"If our school ever gives up the strict inductive method of Hahnemann, we are lost, and deserve only to be mentioned as a caricature in the history of medicine."—CONSTANTINE HERING.

Vol. XVIII. AUGUST, 1898. No. 8.

### OFFICIAL ACCOUNT OF THE EXHUMATION OF THE BODY OF HAHNEMANN AT THE CEMETERY MONTMARTRE AND OF ITS REMOVAL TO PERE-LACHAISE.

Furnished by the French Homœopathic Society and the Representatives of the International Tomb Committee, present at the ceremony on May 24th, 1898.

[The hearts of all homœopathists whose eyes rest on these pages, whether patients or physicians, will throb in sympathy with the pathos and enthusiasm which they portray. The glowing words of Dr. Cartier and Dr. Léon-Simon paint before our minds a vivid picture of the solemn but simple and touching ceremony around the grave of him whom we are proud still to call our Master. We are proud, for, great as is the truth of Homœopathy, Hahnemann taught us even more than this. He taught us to observe, to experiment, to judge for ourselves.

The new spring of life described by our article carries a lesson with it—a type of youthfulness and of resurrection. We think of Hahnemann in his young days—a scholar, for at twenty we are told he was acquainted with Hebrew, Greek,

Latin, French, Italian, English, and perhaps Arabic; a scientist (witness his varied writings on chemistry, hygiene, etc., in the best medical journals of the day); and a physician. In these, his pre-homœopathic days, he was successful, sought after, and honored, at Dresden, Leipsic, and Mayence. Good had it been for Hahnemann had he never been a physician—but bad for his posterity. We think of him paying dearly for his grand discovery—persecuted and driven from Königslutter, from Hamburg, from Leipsic, and, skipping over his period of rest and success in Coethen and Paris, we follow him to his obscure and unnamed grave in Montmartre Cemetery. Is it too much to say that he spent his long life for others—for humanity rather than for himself; that he lived and died a martyr to the convictions and teachings to which we owe so much? But as time removes the prejudice against which he struggled, history begins to record the verdict of posterity. If he died unhonored, he lives again in tens of thousands of his followers all over the world, and millions of sufferers have arisen to bless his name. Untold numbers will rejoice that his grave has not been allowed to pass into oblivion, but that with the growth of his fame and the spread of his doctrines the age demands that justice be done to him. No longer unhonored, he takes his place among the departed heroes of that great nation which gave shelter to him in his later years. The spring time has arrived for him once more.

In the name of our confrères in this country and of their many patients—Hahnemann's debtors and admirers—we congratulate our colleagues in Paris on the energy and loyalty they have shown in the work intrusted to them by their brethren the world over. We are thankful for the completeness of their success, and in particular our gratitude is due to Dr. Cartier, on whose unwearied devotion so much has devolved. We look forward to the great gathering which shall take place in Paris in 1900, when representatives from all parts of the globe will pay the grateful homage of all nations to the genius, the labors, and the altruism of Samuel Hahnemann. Perhaps it is too much to hope that even those who fail to understand and follow him will have the liberality, good taste, and judgment to join in honoring him so soon. But this will come—and more also.

The subjoined report is translated from the official account published in the *Revue Hom. Francaise*.—EDS. *M. H. R.*]

On Tuesday, May 24th, 1898, in the presence of the civil authority and of thirty-five other persons, the solemn exhumation of the body of Samuel Hahnemann, founder of Homœopathy, took place. The ceremony began at half-past eight in the morning by the arrival of the Commissioner of Police, representing the civil authorities, who permitted the exhumation of the body of Hahnemann and that of his wife, in accordance with the arrangement made with the Baroness Bœnninghausen, the adopted daughter and heir of Hahnemann's widow. There were present:

Dr. Süss Hahnemann, grandson of Samuel Hahnemann, who came from England.

Mr. Cloquemin, Vice-President of the Transatlantic Company, representing the Baroness of Bœnninghausen.

The International Committee was represented by Dr. Richard Hughes, of Brighton, and by Dr. François Cartier, Secretary of the Paris Committee.

There were present also the following doctors and chemists: Léon-Simon, President of the French Homœopathic Society; Parenteau, Conan, Jousset, Sr., Jousset, Jr., Nimier, Faure (J. B.), Guinard, Faure (Elie), Tissot, Dezon, Nuguay, Boyer, Love, Chancerel, Sr., Chancerel, Jr., Georges Tessier, Trichon, Peuvrier, Heermann, Vautier, Koenick, Giradeau, Ecalle, and Bernard Arnulphy, of Chicago.

Dr. Gannal, who was present at the embalming of the body of Hahnemann as an assistant to his father fifty-five years ago, was also present at the ceremony. Thirty-five persons in all gathered for the occasion, including five who were not medical men.

A telegram from Dr. De Brasol, President of the Committee, addressed to the Secretary of the Committee, was read at the beginning of the ceremony:

"Am not able to come, am with you in spirit at Paris, taking a deep interest in this solemn occasion. It is comforting that due honor is at last being rendered to our master. Wish success to the work you have so energetically undertaken, and that in two years the tomb may be adorned by the beautiful monument.—DR. BRASOL."

Dr. Cartier was the first to speak:

"Gentlemen—In face of this open grave and before this coffin containing the body of Samuel Hahnemann, our illustrious master, my duty is not to retrace the work of this man of genius, who has moved the world by his ideas and his doctrines. As Secretary of the International Committee of this monument and French delegate, the only one able to act on the spot, I must give to you who are present here and to all those who over the whole world are anxiously awaiting the result of to-day's ceremony, clear and decisive proof that we are in the presence of the precious remains of Samuel Hahnemann, and that the monument that we are to erect at Père-Lachaise will undoubtedly cover the body of the founder of Homœopathy. This is necessary, because of recent polemics which have appeared on this subject in different homœopathic journals; and which it is very important to correct by giving authentic proofs.

"The evidence may be summed up in two groups:

"First. The information furnished by the register of the civil authority, and by the statements of the family and of homœopathists coinciding with the marks on the vault and on the coffin.

"Second. And finally opening of Hahnemann's coffin, whose features ought to be still recognizable. We have to demonstrate that Hahnemann was buried in the grave of Lethière, and that it is Hahnemann's body which is found on the opening of the first coffin. On the one hand, the books of the cemetery and of the registrar; on the other, the information furnished by the grandson of Samuel Hahnemann, Dr. Süss Hahnemann, who is here present; by Madame Bœnninghausen, the adopted daughter of the late Madame Hahnemann, née d'Hervilly, by Hahnemann's contemporaries, by those who have written on his life; all these testify that Christian Samuel Hahnemann died in Paris in 1843, and was buried in Lethière's sepulchre, indicated by a perpetual grant, bearing the No. 324, of 1832, and 414 of 1834. On the left is the Hahnemann sepulchre, bearing the No. 231, in the year 1847.

"This sepulchre contains only the body of Hahnemann's widow, née Mélanie d'Hervilly, who died in 1878.

"Certain homœopaths have erroneously supposed that the body of Hahnemann was put into this sepulchre.

"Gentlemen, it is now open before you, it contains only one coffin, whose description answers to the registered account of Madame Hahnemann, née d'Hervilly.

"The sepulchre Lethiére, where the body of Hahnemann reposes, has been reproduced in a print, in the journal of Dr. Schwabe, the *Homœopathischer Kalendar*, in 1892, more recently in the *Hahnemannian Monthly* of October, 1896.

"Since the drawing was made of the sepulchre, the zinc roof has been taken away, but you can see its identity with the drawing that I show you, by the iron railing and the shape of the tombstone. Finally, you see, as a convincing proof, in the corner of the tombstone, this inscription: 'C. P. 324.' (Perpetual Contract 324.) We knew also, through the cemetery authorities and through the account of the family and homœopathic physicians, that Hahnemann's coffin was the last interred. The body of Gohier was the first buried, the cemetery authorities possess no longer the date of his death; the body of Lethière, who died in 1832, is in the middle, then the last one, that is to say the first beneath the stone, is the body of Hahnemann, buried in 1843. The number of

Hahnemann's coffin inscribed in the register of Montmartre Cemetery is No. 1252, 1st ward (*arrondissement*), 1843.

"Now, gentlemen, you are here to-day to verify these facts. We read distinctly on the first lead coffin which presents itself, separated from the others by a layer of cement, immediately below the stone of the Lethière sepulchre, the following inscription, which has not been at all altered by the weather: 'No. 1252, 1st ward (*arrondissement*), 1843.' Higher up, on the coffin, you can see a lead stamp (mark) as follows:

" 'BREVET D'INVENTION.
*Embaumement Gannal.*'

"Now we know that Hahnemann's body was embalmed by one of the first specialists of the day.

"The firm of Gannal still exists, 6 Rue de Seine. I had the opportunity of seeing Dr. Gannal, the son and successor, who was his father's assistant at the embalming of Hahnemann, and who still remembers the operation. The embalming was done, according to him, with sulphate of Alumina (Gannal's process), although Dr. Süss Hahnemann, equally an eye-witness, asserts that Arsenic was the agent employed.

"On Gannal's books these words are still found: 3d July, 1843, 'Embalming of Dr. Hahnemann, 2,000 francs.'

"To-day Dr. Gannal is with us and desires to be present at the exhumation. I will now sum up in order the proofs of the identity of Samuel Hahnemann's body.

"First. Hahnemann was buried in the sepulchre Lethière, and not in the Hahnemann's sepulchre, in accordance with the statement of an eye-witness, Dr. Süss Hahnemann, grandson of Hahnemann, with the attestation of Madame de Bœnninghausen, adopted daughter of Madame Hahnemann, his widow, with the writings of all those who have related Hahnemann's life.

"Second. Hahnemann's coffin, in the Lethière sepulchre, is certainly the one bearing the No. 1252, 1st ward (*arrondissement*), 1843.

"For, firstly, the No. 1252, visible on the coffin, corresponds with the number in the cemetery's register; secondly, the Rue de Milan, where Hahnemann died, at the present time in the 9th ward (*arrondissement*), belonged to the 1st ward of Paris in 1843.

"Third. Hahnemann alone died in 1843, and was buried in the Lethière sepulchre, where two other bodies already lay buried, one in 1832, and the other at a still earlier date.

"Fourth. The stamp bearing the mark of Gannal's embalming is still another proof.

"Finally, gentlemen, in order to dispel any remaining doubts, I obtained from the police authorities consent to open the lead coffin. We are going to be present at a touching and unique spectacle; we shall look upon the remains of him who is our everyday guide—our common master. The features of Hahnemann, who has slept for these fifty-five years, will see the light for the last time."

After the address of Dr. Cartier, M. Cloquemin spoke. On behalf of Madame de Bœnninghausen, whom he represented at the ceremony, he thanked the Homœopathic Society, and in particular Dr. Cartier, for the work of the homœopathic physicians, in which, he said, Madame la Baronne de Bœnninghausen takes the greatest interest. She rejoices to know that the remains of her mother, of whom she entertains a most affectionate remembrance, will be placed with those of Dr. Hahnemann in the same grave in Père-Lachaise.

Dr. Simon, President of the French Homœopathic Society, then gave the following address:

"Gentlemen—Thanks to the good-will of Madame la Baronne de Bœnninghausen, to the kindness of M. Cloquemin, and to the zeal of Dr. Cartier, we are able to honor the memory of Samuel Hahnemann according to our dearest wish; the French Homœopathic Society offers them its sincere gratitude. It is ready to receive the two coffins from the committee represented by Dr. Hughes and Dr. Cartier. You may rest assured that we shall carefully watch over this precious charge. Two generations have already passed, gentlemen, since our master left this world, and to the grandchildren of his contemporaries the unexpected task falls of providing him a tomb less modest than the one in which he has rested until now. Strange turn of events, which proves once more that man proposes and God disposes; which also demonstrates again that Hahnemann's glory is proof against time!

"For firstly he lives again in his grandson, who follows faithfully in his footsteps; and next his name is not likely to be forgotten, because instead of working for his own time and for himself, he has worked for all ages and for humanity.

"Therefore, it is of little importance that the present age, blind and ungrateful, has disowned and disdained him, posterity of which we are the vanguard, is ready to do him justice.

"Hail, Hahnemann! We bow before thy venerated remains, to which, more fortunate than our predecessors, we can render the honors due. Full of faith in the future, we give our brethren rendezvous at thy mausoleum at the Congress of 1900. The tomb will appear to them more glorious, enlightened by the dawn of the next century, which will certainly see the triumph of thy teaching."

After the earnest speech of Dr. Simon, who deeply moved the audience, Dr. Richard Hughes, of Brighton, came forward and gave the following address in French:

"Gentlemen—In obedience to the wish of my colleagues, I say a few words in the name of the English homœopaths, and you will forgive me if I do not express myself as well as I should desire in your language.

"England cannot boast of being either the birthplace or the burial place of Samuel Hahnemann; she is not, however, wanting in her devotedness to his memory, any more than

Germany or France. Her institutions show it. In the year when he died, she had already started the *British Journal of Homœopathy*, and in the following year the British Homœopathic Society was founded. Five years later the London Homœopathic Hospital was opened, lately rebuilt at a cost of £48,000, and containing now a hundred beds. The *Journal* bore up the flag of Homœopathy for forty-two years; the Society and the Hospital continue their work to this day. As a representative of them, and also of our present journals, I come amongst you to-day, bringing their fraternal salutations to *l'Art Medical*, to the French Homœopathic Society, and to the Hahnemann and Saint Jacques Hospitals.

"You have heard from Dr. Cartier what we have to do and what has already been accomplished. Our warmest thanks are due to him, as well as to the Society for which he acts, for having so well cleared away all obstacles from our path. To-day the disciples of our master can reclaim his precious body, look upon his features—so calm in the profound rest of death, and take him out from his obscure surroundings in order to deposit him among the

" 'Kings of thought
Who wage contention with their time's decay,
And of the past are all that cannot pass away.'

"This is our task for to-day. To-morrow we shall make ready to erect over these remains a monument worthy of his merits and of our veneration for him, at the sight of which the world can ask, What was this man, to whom after more than fifty years his disciples have shown so much honor? It will ask; and those who know his worth already will make pilgrimage from all the countries of Europe, from North and South America, from India, from Australia, and will rejoice to see the master thus honored. They will return home, armed with a new courage to follow in the path which he opened up for the advancement of their art and for the benefit of their patients.

"French colleagues! England joins with you in your desires and in your work."

SPEECH OF DR. SUSS HAHNEMANN IN FRENCH.

"As a representative of Germany and of Hahnemann's family, I am very happy that I am allowed to take part in this interesting ceremony. Fifty-five years ago I was present at the funeral of my grandfather, who was left here without a name and without a monument for more than half a century.

"Thanks to the International Committee, and especially to Dr. Cartier, Samuel Hahnemann has found a resting place worthy of his name."

OPENING OF THE COFFIN.

After the speeches were concluded the workmen proceeded to the exhumation of Hahnemann's coffin. In the presence of the Commissioner of Police the workmen raised the coffin to the surface by means of ropes; it was placed on the boards which covered up the hole made by the previous exhumation of Madame Hahnemann. Dr. Gannal, who superintended the operations, discovered that the lead coffin of Hahnemann had been screwed down and not soldered, and he told the physicians that he feared the body might not be well preserved. The workmen removed the screws which were not too rusty, and forced out those which were worn out by age. The lead cover gaped at the end, and those who were present perceived Hahnemann's feet, wrapped up in cloths, resting against the sides of the coffin; they appeared well preserved, but as they continued pulling out the screws, and as the lid opened wider, it was noticed that there was water in the coffin, and the fears that the body would not be well preserved increased.

At last the lid opened wide, and Hahnemann's body was seen, covered and wrapped up with silk bandages. The conformation of the body, outlined under the embalming bandages, was preserved; the body was slightly shrunken, but what most struck the onlookers was the short stature of Hahnemann. On asking those who knew him, we got the reply that the founder of Homœopathy was, in fact, very short. The body was lying in water, the fluid not being produced by the embalming, but coming from the outside. The soil of Montmartre Cemetery was continually infiltrated, according to competent authorities, the water flowing along from the clay bottom; but if the coffin, in 1843, had been so.dered and not screwed, it would not have penetrated. Water in the coffin necessarily brought about the decomposition of the body.

The embalmer took great care, besides applying the silk bandages, to cover the head and the hands with pieces of wool soaked in "essence." At the end of the half-century these pieces of wool appeared like large sponges enveloping Hahnemann's head, and his hands which were crossed over his body.

Dr. Gannal removed from the face and hands the remains of the wool and silk bandages, which were better kept than the rest.

The head was found to be a mere mass of decomposed tissue and bones. He searched for the glass eyes which had had to be placed in the orbits.

Hahnemann's body was completely decomposed. There only was found a long tress of woman's hair twisted round the neck, probably Madame Hahnemann's hair.

In view of its being an impossibility to recognize Hahnemann's features, Dr. Gannal was fortunately able to produce for us several tokens from the coffin, which assured the identity of the body, and which we give in detail.

FIRST.—THE WEDDING RING.

Dr. Gannal examined the separated bones of the hand, and finished by discovering on one of the metacarpals Hahnemann's wedding ring. This gold ring was shown to the spectators; it was made of two small ones, which could be separated by a penknife, and on one of them was engraved these words:

"*Samuel Hahnemann. Melanie d'Hervilly.*
*Verbunden Coethen, 18 Janvier, 1835.*"

The ring was replaced on one of the bones of Hahnemann's hand by order of the Commissioner of Police.

SECOND.—THE GOLD MEDAL FROM THE FRENCH HOMŒOPATHS.

At Hahnemann's feet was found a bottle corked with emery and sealed up. The police officer gave permission to break it; it contained papers respecting Gannal's process of embalming, the gold medal from the French homœopaths to their master, together with an autograph letter from the late Madame Hahnemann, which formed the final link in the chain of evidence of identity furnished by the coffin. The gold medal, in an excellent state of preservation, represents on one side Hahnemann's profile, by David of Angers, the sculptor of Hahnemann's famous bust, which is used as a model for his portraits. On the other side is the following inscription:

"*A leur maitre, les homœopathistes Francais.*
*Similia Similibus Curantur.*"

This medal was struck in bronze. Dr. Boyer had brought with him an exactly similar specimen to that found in the coffin. After having been examined by the company the gold medal was replaced therein.

THIRD.—THE AUTOGRAPH OF THE LATE MADAME HAHNEMANN.

Among the papers concerning the embalming, found stored in the bottle, was an autograph letter from Madame

Hahnemann, which the Commissioner of the Police permitted to be reproduced by photography. The authenticity of the handwriting of Madame Hahnemann was attested by witnesses who had known the widow of the discoverer of Homœopathy. Monsieur Cloquemin, representing the Bœnninghausen family, and Dr. Heermann (of Paris), recognized the handwriting without the least hesitation.

The facsimile of the letter is here reproduced:

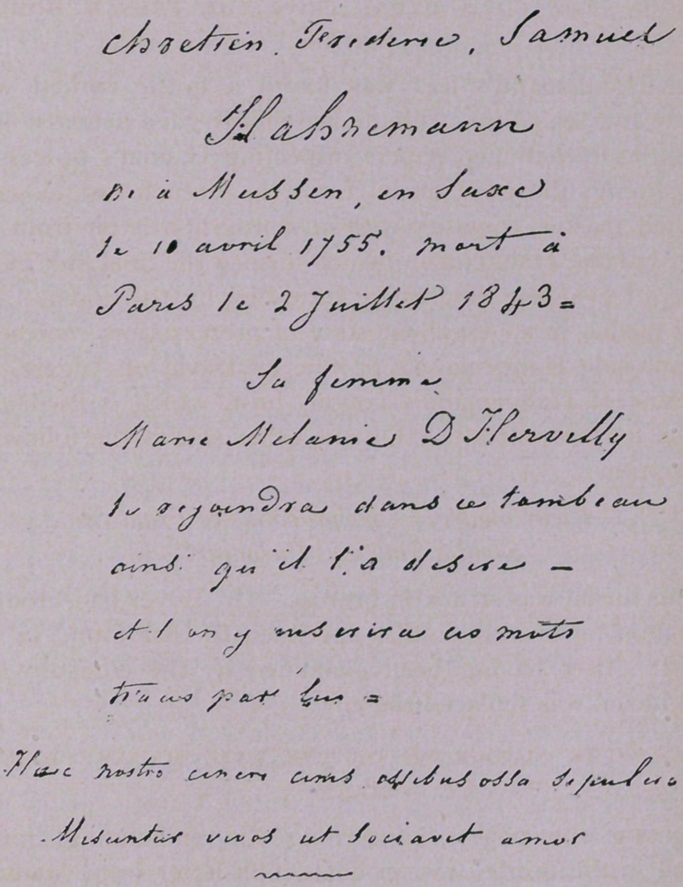

Chretien. Frederic, Samuel
Hahnemann
né à Meissen, en Saxe
le 10 avril 1755. mort à
Paris le 2 Juillet 1843 =
Sa femme
Marie Melanie D'Hervilly
le rejoindra dans ce tombeau
ainsi qu'il l'a desiré —
et l'on y inscrira ces mots
tracés par lui =
Haec nostro cineri cinis ossibus ossa sepulcro
Miscentur vivos ut sociavit amor

### END OF THE CEREMONY AT MONTMARTRE.

By ten o'clock the ceremony at the Cemetery Montmartre was over, having lasted an hour and a half. The workmen replaced the lead cover; the leaden shell was then put into a new wooden coffin, on which they nailed the old plate (No. 1,252, 1st ward, 1843), also a very large new copper plate on which the name "Samuel Hahnemann" was engraved. At this time the company withdrew, convinced of the identity of the body, but regretting the unsuccessfulness of the embalming.

Hahnemann's coffin, and that of his wife, were placed in a hearse, and ten persons accompanied it to the Cemetery of Père-Lachaise, among whom were Drs. Süss Hahnemann, Richard Hughes, Simon, Heermann, and Cartier, and Monsieur Cloquemin.

### AT PERE-LACHAISE.

By contrast with the retired spot in Montmartre, so small and mean, the new resting place of Hahnemann appears a veritable rehabilitation. The Chemin du Dragon at Père-Lachaise, where the founder of Homœopathy is now buried, is one of the most picturesque of roads, planted with a variety of trees, and having about it something at once grand and mysterious. Perhaps this name was given to it on account of a likeness to the places where this mysterious and incomprehensible creature was supposed to frequent. At every turn in this renowned corner of Père-Lachaise the mind lives again with all the grand and celebrated men that France has sheltered in science, the fine arts, and war. Here music is represented by Rossini, Auber, Donizetti; there the poets and celebrated writers. Racine lies almost beside Hahnemann; a little further on are Molière and Lafontaine. Science is represented by Gay-Lussac and Arago. The celebrated physician and neurologist, Gall, is a few steps lower down than Hahnemann. Lastly, on the same side are the tombs of the marshals of the First Empire—Ney, Davout, etc. The Chemin du Dragon is the route usually taken by tourists who visit this renowned cemetery—the chief in Paris—by thousands.

"What good fortune!" exclaimed one of the company, on arriving at this place. In fact, Hahnemann's tomb is just on the border of the Chemin du Dragon where two roads cross.

In this way the site of the future monument can be reached by three different routes.

Whilst the spectators admired this part of the cemetery and its adornment of spring verdure, the gravediggers put Hahnemann's coffin into the grave. They placed the body parallel with the road, in such a manner that the head of the great man will be found at the right-hand side of the monument, the feet at the left; finally, the little coffin enclosing the remains of Madame Hahnemann was placed at Hahnemann's feet. The workmen immediately proceeded to cement the vault after putting down the two coffins, and covered them with concrete in the presence of the onlookers, who only left the place after the grave had been perfectly closed and filled up.

A temporary railing and a crown will be the simple ornaments over the precious remains of Hahnemann, until the day when, deeply moved, the homœopathists from all parts of the world will complete the work of restoration in honor of their venerated master—a work the more brilliant because so long deferred.—*Monthly Homœopathic Review*, of London, England. Edited by Drs. Pope, Dyce Brown, and E. A. Neatby. July, 1898, page 385.

